# Impact of Teacher's Mental State Talk on Young Children's Theory of Mind: A Quasi-Experiment Study

**DOI:** 10.3389/fpsyg.2021.668883

**Published:** 2021-03-26

**Authors:** Jianfen Wu, Minmin Liu, Wenqi Lin

**Affiliations:** ^1^School of Education, Hangzhou Normal University, Hangzhou, China; ^2^Hangzhou Qiantang Jiangchao Kindergarten, Hangzhou, China

**Keywords:** theory of mind, mental state talk, teacher-child interaction, young children, quasi-experiment

## Abstract

This study investigated the relationship between teachers' mental state talk and young children's theory of mind with a quasi-experiment. In total, 56 young children were assigned to the experiment group (mean_age_ = 41 months, SD = 2.47, 46% girls) and the control group (mean_age_ = 40.68 months, SD = 2.23, 43% girls). The experiment group was engaged in a 12-week intervention program with mental state talk in storytelling, casual conversations, and role-playing games, whereas the control group received no interventions. All the children were tested with three theory of mind (ToM) tasks before and after the intervention. The results indicated that the experimental group had a significant improvement in the ToM scores, whereas the control group showed no significant change. The educational implications of these findings are discussed.

## Impact of Teacher's Mental State Talk on Young Children's Theory of Mind: a Quasi-Experiment Study

Young children can usually pass false belief tasks and acquire a mature theory of mind (ToM) by age 5 years (Carr et al., 2018). This is because parents' use of mental state language plays a direct and causal role in the development of ToM in young children (Devine and Hughes, 2018, 2019). However, most young children will enroll in preschools or kindergarten from age 2 years, transiting into a more diverse and varied social context: the preschool classroom. In the classroom, they need to interact with peers and teachers who might use mental state talk to facilitate their ToM. So far, few studies have explored the role of teacher's mental state language in the early development of ToM. To fill this research gap, this preliminary study adopted a quasi-experiment design to examine the effect of teacher's mental talk on young children's ToM development.

### Meta-Language, Mental State Talk, and ToM

ToM refers to an individual's cognition and ability to attribute mental states to ourselves and others, serving as one of the foundational elements for social interaction (Devine and Hughes, 2018, 2019). In particular, mental states mainly include an individual's belief, needs, desire, intention, feeling, knowledge, and emotion (Happé et al., 1998). As a core component of the naive theory, ToM is an effective tool for children to understand society. Having a ToM is important as it empowers young children to predict and interpret the behavior of others. With ToM, children would have a better understanding of others and, as a result, can better adapt to social life (Carr et al., 2018). Therefore, ToM plays an important role in early social development and thus has been empirically studied by developmental scholars. In the past decade, researchers have primarily focused on the developmental patterns and individual differences of children's ToM using different tests. Later on, the research focus has been shifted to parental influences, especially mothers' mental talk during the mother–child interactions. This is because early social interactions could promote children's ToM development; mother–child interaction is one of the most important ones in the early years (Feldman, 1992). Therefore, the existing studies have jointly indicated that parental mental state talk could significantly facilitate the development of ToM in young children (Carr et al., 2018; Devine and Hughes, 2018, 2019). However, social interaction experiences have two interrelated components in the early years: family interaction experiences and preschool interaction experiences. So far, few studies have explored the impact of teachers' mental state talk on young children's ToM development, which is exactly the research gap to be filled by this study.

In the past two decades, an increasing volume of research has been focused on the relationship between meta-language and ToM. Meta-language is a language that relies on terms describing and presenting the mental state in the communication, which can promote the development of children's social cognition and ToM. Generally, mental state term refers to those about belief, desire, intention, emotion, and perception; narrowly speaking, it can refer to those cognitive terms such as think, know, guess, and remember (Milligan et al., 2007). The existing studies found that mental state terms such as “want,” “know,” “think,” and “remember” could be produced by children aged 2–3 years (Shatz et al., 1983; Tardif, 2006). However, young children might not necessarily use these mental state terms to express their mental state; instead, they might use them as a measure of communication (Shatz et al., 1983). Even though this use in daily communication could promote the development of children's ToM, as it can enhance their semantic cognition of verbs such as “want” and “know,” a kind of application of mental state terms (Astington, 2000). To precisely and correctly express mental states, young children need to understand their beliefs and mental states. Therefore, many scholars tend to regard mental state talk as a milestone in children's understanding of various mental states of themselves and others when using mental state terms such as “want,” “think,” “know,” and “remember” in their talk (Shatz et al., 1983; Lewis and Mitchell, 1994).

Recently, some studies have examined the effectiveness of early language activities such as storytelling on promoting mental state talk (Ornaghi et al., 2011). Children can understand the content by reading or listening to the story and then answer the related or follow-up questions related. This activity can detect whether young children can understand the content of the story and the meaning of the mental state terms contained in the questions. Young children need to choose the best mental state terms to describe their mental state in the story scenario. A very recent study in China found that if young children listened to stories containing rich mental state terms and conducted dialogue training on common mental state terms, they would significantly comprehend false beliefs, emotional beliefs, and mental state terms (Zhang, 2018). However, few studies have explored the impact of teacher's mental state talk on the development of ToM in young children. Therefore, this study is dedicated to filling the research gap by conducting a quasi-experiment study.

### Teacher's Talk and Young Children's ToM

Few studies have examined the relationship between teacher's talk and young children's ToM. First, one project-based study found that the teachers with mental state talks could facilitate young children's understanding of themselves and others (Frampton et al., 2009). Later, another study found a significant positive correlation between the number of mental state terms in teacher–children conversations and the development of children's ToM (Lecce et al., 2014). A follow-up study found that conversations about the mind effectively enhanced ToM during middle childhood (Bianco et al., 2016). Meanwhile, Chinese scholars have also conducted a study on the development of preschoolers' mental state cognition ability and teacher–student discourse quality, which explored the structure and quality of teacher–child verbal interaction and their impact on children's ability to understand others' mental states (Qiu et al., 2015). A very recent study in China found that the extensive use of teachers' mental state terms or the extensive use of a certain syntactic structure would be conducive to improving children's ToM (Zhang, 2018). However, it should be noted that most of these studies were exploring the relationship between parent–child interaction and a child's ToM development. So far, no studies have explored the effectiveness of preschool training programs on the development of ToM in Chinese young children. As an important context for early development, preschool classrooms provide great opportunities for teacher–child interactions and thus have the potential to contribute to the ToM development.

Accordingly, this study designed and implemented a set of early ToM training programs and examined its effectiveness in promoting ToM in Chinese preschooler. To increase its ecological validity, we adopted the three typical preschool activities such as storytelling, casual conversations, and role-play activities. Thus, mental state talk has been deliberately incorporated and elaborated in the experimental group program, whereas the control group had similar activities without any mental state talk. We hypothesized that the experimental group would perform significantly better after the training intervention than their counterparts in the control group. In particular, the following questions guided this study:

1. Did the experimental group outperform the control group significantly in the pretest of ToM before the early intervention?
2. Did the experimental group outperform the control group significantly in the posttest of ToM after the early intervention?

Accordingly, we tested the following hypotheses in this study:

***Hypothesis 1***: The experimental and the control groups should have no significant differences in the pretest of ToM before the early intervention;

***Hypothesis 2***: The experimental group would significantly outperform the control group in the posttest of ToM after the early intervention.

## Methods

### Sample

One public kindergarten in Hangzhou, Zhejiang Province, consented to participate in this study. All the parents of the reception classes (aged 3–4 years; *N* = 148) were invited and consented to participate in this intervention study. All the children were administered with ToMs in the pretest, and 56 of them scored below the mean level thus were randomly assigned to either the experiment group (*n* = 28) or the control group (*n* = 28). The two groups did not differ significantly on variables such as age, gender, and index of ToMs (Table 1). All the 56 participants lived in the neighborhood, and their parents worked either in local factories or companies. Approximately 39 parents had an associate degree (35%), 62 had bachelor's degrees (56%), and 11 had master's or doctoral degrees (10%). The annual family income median is between 150,000 and 200,000 Chinese Yuan (RMB). The children in both groups were not clinically referred for any cognitive or learning difficulties. The Independent *t*-test indicated no significant difference between the two groups (*p*'s > 0.05), indicating that the two groups were homogeneous before the intervention.

**Table 1:** Descriptive statistics for the experimental and control groups in pretest.

	**Experiment group *n* = 28**	**Control group*n* = 28**	***t***
Gender	15 boys, 13 girls	16 boys,12 girls	
Age (month)	41 (2.47)	40.68 (2.23)	
Age range (month)	36–48	36–47	
Unexpected location task in pretest	0.57 (0.69)	0.75 (0.70)	−0.96
Unexpected content task in pretest	0.93 (0.47)	0.89 (0.63)	−0.24
Appearance–reality task in pretest	0.57 (0.50)	0.43 (0.50)	1.06
Total ToM score in pretest	2.07 (1.05)	2.07 (1.09)	0.00

And the teachers of both groups had no significant differences in qualifications and teaching experiences. All of them held a bachelor of education degree majoring in early childhood education. In the experimental group, the class teacher had 5 years of teaching experience, and the assistant teacher had 1 year of teaching experience. In the control group, the class teacher had 3 years of teaching experience, and the assistant teacher had 4 years.

### Instrument

Preschooler's ToM was tested, before and after the intervention, using the ToM unexpected-location task (Bartsch and Wellman, 1989), the ToM unexpected content task, and the appearance–reality task. Each task should take <10 min, resulting in <30 min for each child. The total score for the tasks ranged between 0 and 5.

***ToM Unexpected Location Task***(Perner et al., 1994). In this task, a female doll was named “the elder sister,” a male doll was named “the younger brother;” there was a yellow backpack with a cartoon pig's image, a shoebox with a lid, and a toy car. First, a story was presented to the child, accompanied by moving the toys to aid comprehension: Scenario 1: “The elder sister and the younger brother are in a room. The elder sister has a shoebox with a lid. The young brother has a backpack with toy car inside.” Scenario 2: “The younger brother leaves the room.” Scenario 3: “The elder sister takes the toy car and puts it in her box.” Scenario 4: “Now the younger brother comes back. He wants his toy car.” The child is then asked the following questions: “Where will the younger brother look for his toy car? Why?” The child was scored 0 if he or she answered the question incorrectly or was unable to justify the answer, and 1 if he or she answered and explained correctly. The total score for this task ranged from 0 to 2.

***ToM Unexpected Content Task***(Perner et al., 1987). The child was shown the box “Goldfish” (a type of cookie) and asked what they thought was inside. After they predicted the typical content (cookies), the box was opened to show them the atypical content (colored pencils). The pencils were placed once again in the box, and the test questions were asked about both the child's own belief before seeing the atypical content and about another's belief (classmate). The order of these two questions was counterbalanced. Children were scored 1 point for each of the two questions if they answered correctly. The total score ranged between 0 and 2.

***ToM Appearance–Reality Task*** (Gopnik and Astington, 1988). First, the child was shown a sponge that looked like a stone, and the experimenter asked, “What do you think this is?” If the child replied “a stone,” he or she would be allowed to touch the object to see that it was, in fact, a sponge. Next, the child was asked the false belief questions about themselves such as “What did you think it was before you touched it?” and about others “When XXX (classmate) comes in and is shown this, without being allowed to touch it, what will he or she think it is?” In this study, the order of the questions was counterbalanced for the participating children. The child was scored 1 point for each of these two questions if he or she answered correctly, and their total scores ranged between 0 and 1.

### Procedure

#### Ethical Clearance

This study was reviewed and approved by the first author's university. An invitation letter was sent to the participating kindergarten, and the principal and the class teachers consented to participate in this study. All the parents were briefed about this study and consented to allow their children to participate in this study. Altogether, all the participating parents, teachers, and child-care workers signed written consent for this study. The participating young children verbally agreed to attend this study, knowing that they have the freedom to deny evaluation or withdraw from this study at any time.

#### Four Stages

The study consisted of four phases: pre-experiment, pretest, training, and posttest. First, pre-experiment was conducted a week before the experiments formally started. Pre-experiment helps the experimenters familiarize themselves with the testing procedures through rehearsals and make appropriate adjustments to the training program. All eight preschoolers (including four girls) completed the entire pre-experiment procedures. Consequently, the training materials and instructions were finalized based on the preschooler's feedback. These eight preschoolers would not participate in the formal experiments in the main study. Second, all the children were administered the three tasks in the pretest. Third, training was initiated 2 weeks after the pretest phase ended. Fourth, the intervention lasted 12 weeks. Last, the posttest phase took place 1 week after the end of the training. The same set of ToM tasks was adopted in the pretest and posttest phases to minimize the statistical deviation and the effects that such deviation might cause. In addition, this experiment used a relatively balanced method to test the sequence of tasks and randomly used cross-coding to conduct the validity analysis.

#### Intervention Design

The 12-week school-based mental state talk intervention program was designed and implemented in this study. The experimental group children attended 24 story activity sessions held twice weekly, 120 conversations held 10 times weekly (twice daily while there are five school days a week), and 48 games held four times weekly. The details of the intervention program are as follows.

##### Storytelling

It lasts 40 min. Preschoolers aged between 3 and 4 years are mostly self-centered and rarely pay attention to the emotions and feelings of people around them. Teachers may apply storytelling as a teaching method to lead children to recognize various emotions, such as happiness and sadness so that children can perceive the meaning of other people's facial expressions. For example, “He and his mother are separated, and now he is alone and lonely. Do you think he is happy?” By using the characters in the story as an object, teachers can have a dialogue with preschoolers on these characters' different mental states. Consequently, preschoolers are guided to be considerate toward other people's points of view.

##### Casual Conversations

They last 10 min. Teachers may guide children to understand the mental state of others, which helps to cultivate their empathy. For example, when talking about a child who accidentally falls onto the ground, teachers and preschoolers can start a casual conversation activity about the topic of “falling.” The teacher could ask preschoolers: “How do you feel when you fall?” and “How do other children feel when they fall?” Afterward, teachers ask preschoolers to describe the mental state of themselves and others and then guide them to develop their empathy gradually.

##### Role Play

It lasts 15 min. Teachers organize preschoolers to carry out role plays. Preschoolers will perform role plays based on their personal preferences while analyzing their characters' minds during the process.

## Results

Statistical analyses were conducted using SPSS (version 20). Descriptive results were presented in Tables 1, 2, indicating the means and standard deviations for all the study variables.

**Table 2:** Means and standard deviations for the ToM tasks in pretest and posttest.

	**Pre-test**		**Post-test**		**Gain scores**	
	**Experiment**	**Control**	**Experiment**	**Control**	**Experiment**	**Control**	**T test**
Unexpected location task	0.57 (0.69)	0.75 (0.70)	1.68 (0.48)	1.11 (0.42)	1.11 (0.63)	0.36 (0.61)	4.49^***^
Unexpected content task	0.93 (0.47)	0.89 (0.63)	1.39 (0.63)	0.93 (0.66)	0.46 (0.64)	0.04 (0.33)	3.16^**^
Appearance–reality task	0.57 (0.50)	0.43 (0.50)	0.75 (0.44)	0.18 (0.39)	0.18 (0.77)	−0.25 (0.44)	2.55^*^
Total score	2.07 (1.05)	2.07 (1.09)	3.82 (0.72)	2.21 (0.83)	1.75 (1.24)	0.14 (0.89)	5.38^***^

* *p < 0.05*,

** *p < 0.01*,

*** *p < 0.001*.

### Testing Hypothesis 1

To confirm whether the experimental and the control groups significantly differed in the pretest (hypothesis 1), we conducted paired-samples *t*-tests on the scores of all ToM tasks. As shown in Table 1, in the ToM unexpected location task, no significant differences were found between the experimental group (mean = 0.57, SD = 0.69) and the control group (mean = 0.75, SD = 0.70), *t* = −0.96, *p* > 0.05. In the ToM unexpected content task, no significant differences were found between the experimental group (mean = 0.93, SD = 0.47) and the control group (mean = 0.89, SD = 0.63), *t* = −0.24, *p* > 0.05. In the appearance–reality task, no significant differences were found between the experimental group (mean = 0.57, SD = 0.50) and the control group (mean = 0.43, SD = 0.50), *t* = 1.06, *p* > 0.05. In the ToM total scores, no significant differences were found between the experimental group (mean = 2.07, SD = 1.05) and the control group (mean = 2.07, SD = 1.09), *t* = 0.00, *p* > 0.05. In summary, all these *t*-test results jointly indicated that there were no significant differences between the experimental and control groups, providing empirical evidence to support hypothesis 1.

### Testing Hypothesis 2

To confirm whether the experimental and the control groups significantly differed in the posttest (hypothesis 2), we conducted paired-samples *t*-tests on the scores of all ToM tasks. As shown in Table 2, in the ToM unexpected-location task, significant differences were found between the experimental group (mean = 1.68, SD = 0.48) and the control group (mean = 1.11, SD = 0.42), *t* = 4.74, *p* < 0.001. In the ToM unexpected content task, significant differences were found between the experimental group (mean = 1.39, SD = 0.63) and the control group (mean = 0.93, SD = 0.66), *t* = 2.69, *p* < 0.05. In the appearance–reality task, significant differences were found between the experimental group (mean = 0.75, SD = 0.44) and the control group (mean = 0.18, SD = 0.39), *t* = 5.14, *p* < 0.001. In the ToM total scores, significant differences were found between the experimental group (mean = 3.82, SD = 0.72) and the control group (mean = 2.21, SD = 0.83), *t* = 7.71, *p* < 0.001. In summary, all these *t*-test results jointly indicated that there were significant differences between the experimental and control groups in the posttest, providing empirical evidence to support hypothesis 2.

### Testing the Training Effects

**Figure 1:**
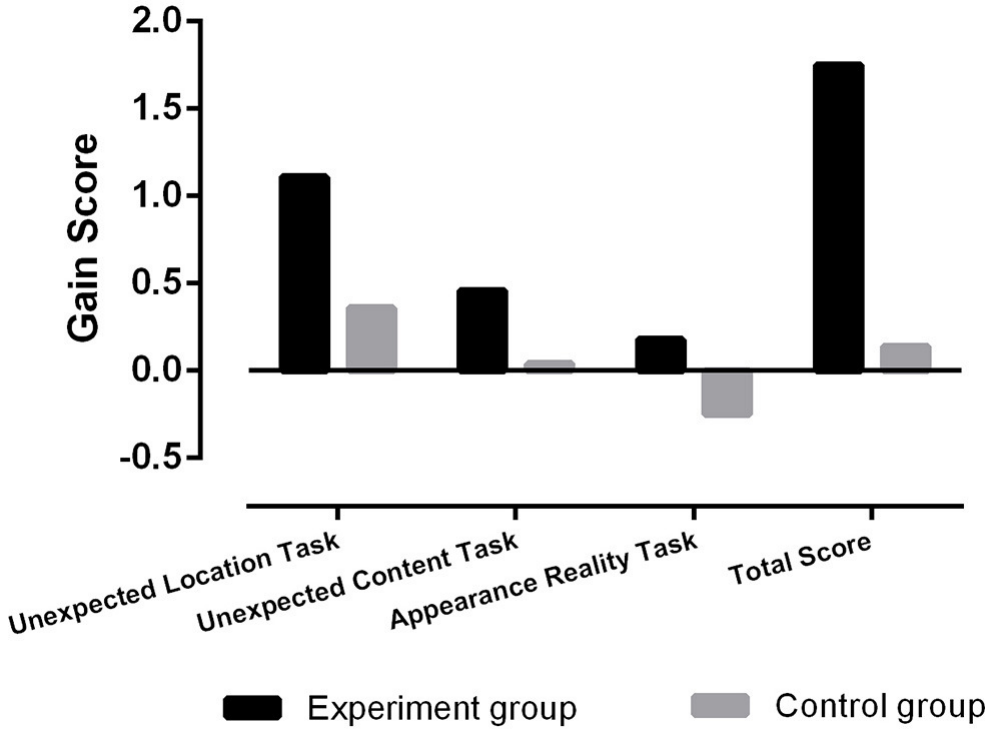
Comparison of gain scores in the ToM tasks between the experiment and control groups.

To compare the training effects of the intervention program, we calculated the gain scores between posttest and pretest for each child. As shown in Table 2, in the ToM unexpected-location task, significant differences were found in the gain scores between the experimental group (mean = 1.11, SD = 0.63) and the control group (mean = 0.36, SD = 0.61), *t* = 4.49, *p* < 0.001. In the ToM unexpected content task, significant differences were found in the gain scores between the experimental group (mean = 0.46, SD = 0.64) and the control group (mean = 0.04, SD = 0.33), *t* = 3.16, *p* < 0.01. In the appearance–reality task, significant differences were found in the gain scores between the experimental group (mean = 0.18, SD = 0.77) and the control group (mean = −0.25, SD = 0.44), *t* = 2.55, *p* < 0.05. In the ToM total scores, significant differences were found in the gain scores between the experimental group (mean = 1.75, SD =1.24) and the control group (mean = 0.14, SD = 0.89), *t* = 5.38, *p* < 0.001. In summary, all these *t*-test results jointly indicated that there were significant differences in gain scores between the experimental and control groups, providing a significant training effect of the intervention program (Figure 1).

## Discussion

As the first empirical exploration of the impact of mental state talk on Chinese preschoolers' ToM, this study designed, implemented, and evaluated a set of early *ToM* training programs. The statistical results found no significant differences between the experimental and control groups in the pretest but significant differences in the posttest and the gain scores, supporting both hypotheses 1 and 2. This section will discuss these findings.

### The Training Effect of Mental State Talk on ToM

This study found that the young children in the experimental group differed from their counterparts in the control group in the posttest ToM scores and the gain scores. This result has confirmed the effectiveness of teacher's mental state talk on young children's ToM. Moreover, the amount of mental state terms that teachers use is significantly higher than the amount used by children's parents (Ziv et al., 2014, 2015). Why can teacher's mental state talk have such a positive impact on young children's ToM? First, during the teacher–child interaction, if teachers consciously use more mental state terms and talk more about their mental state, this will not only demonstrate the correct and precise presentation of personal mental state but also provoke young children's interest to understand and present their mental state (Nelson and Fivush, 2020). The preschoolers' ToM is largely influenced by the mental state terms they use when communicating with others (Dunn et al., 1991). When preschoolers express their mental state, if they cannot find the corresponding words to express, they will not present their own and other people's cognition of their mental state. As a result, they would not share all kinds of information, including experiences, expectations, and ideas, with the people around them (Nelson, 1996; Gopnik and Wellman, 2010). Second, during the teacher–child interaction, teachers ask more about young children's beliefs and mental state, which will inevitably promote their thinking and expression of mental state. Third, storytelling, casual conversations, role-play activities have been employed to deliver mental state talk in the training program. This interesting and natural input of mental state language will help enrich preschoolers' vocabularies for describing their feelings and mental states. Accordingly, they will imitate and try out those mental state terms in the teacher–child interactions. Eventually, it would significantly improve the ToM in the experimental group. This study has preliminarily confirmed the effectiveness of this mental state talk.

### Educational Implications

The finding of this study has some implications for practical improvement in early childhood education and teacher education. First, mental state theories and mental state talk should be included in early childhood teacher education programs. If early childhood teachers know more and present more about mental state, their children will have better development in the ToM. For instance, the following terms should be provided to early childhood teachers in the teacher education programs: (1) terms are used to describe desire such as want, like, and love; (2) terms are used to describe emotion such as happy, sad, and depressed; (3) terms are used to describe affirmation such as maybe, probably, and possibly; and (4) terms are used to describe thoughts such as feel, want, and know (Zhang, 2018).

Second, early childhood teachers should promote young children's understanding of mental state by using more mental state terms. In particular, teachers can create more educational scenarios and discussions to scaffold and promote their learning, understanding, and mental state terms. For example, teachers may demonstrate using “I think,” “I thought,” “I feel,” “I believe,” and “I hope” to describe their feelings of themselves or others. They can also talk about their own or others' opinions and ideas about the events around them and the characters in storybooks. All these trainings will train preschoolers to make predictions about others' innermost feelings and behaviors (Chen, 2018). In the long run, preschoolers would gradually try to consider others' perspectives, views, and feelings and will also care about others' emotions and learn how to handle the differences. This would allow them to predict and explain others' behavior and respond with reasonable feedback based on their perception of others' mental state (Tompkins et al., 2018).

Third, stories, conversations, and role-play activities should be well-used to facilitate young children's ToM. Many stories have content relevant to the mental state, such as desire, knowledge, false belief, surprise, selflessness, hypocrisy, and virtue. Teachers can use more terms like “guess,” “feel,” “know,” “think,” “believe,” and “plan” in storytelling, teaching dialogues, and casual conversations. Teachers can also post more questions about the story characters' thoughts and actions, directing young children to think more about others' thoughts and emotions, which is beneficial to the development of preschoolers' ToM. Many questions can reflect their thoughts, such as what they know, what they expect, what they remember, and what they decide. In addition, teachers can often use some rhetorical questions to promote young children's reflection in casual conversations. Teachers can conduct role-play activities to help young children predict and understand other people's mental states and adjust their behaviors.

### Limitations and Future Directions

This study has some limitations that could be addressed in future studies. First, the sample size is very small in this study. In the future, the sample size should be increased, and the age range could also be expanded. Second, control conditions with general teacher–child interaction without mental state talk could be applied, so as to further exclude the possible effect of teacher–child interaction. Third, this study adopted only three ToM tasks to measure young children's ToM. In the future, some interesting tasks could also be used, such as emotional cognitive and ToM picture books.

## Data Availability Statement

The raw data supporting the conclusions of this article will be made available by the authors, without undue reservation.

## Ethics Statement

The studies involving human participants were reviewed and approved by Ethics Committee of Hangzhou Normal University. Written informed consent to participate in this study was provided by the participants' legal guardian/next of kin.

## Author's Note

We are grateful to all the preschoolers and their teachers in the participating kindergartens. We have no conflicts of interest to disclose. We confirm that appropriate consideration has been made to protect intellectual property rights related to this work. There are no known obstacles to this publication in terms of intellectual property rights and publication time. Nevertheless, we confirm that any experimental work involving animals or human patients in this manuscript is conducted under ethical approval from all relevant institutions.

## Author Contributions

Conceptualization and methodology: JW. Validation: ML. Formal analysis and investigation: JW and ML. Data curation: JW and WL. Draft preparation: JW, ML, and WL. Writing and editing: JW. All authors have read and agreed to the published version of the manuscript.

## Conflict of Interest

The authors declare that the research was conducted in the absence of any commercial or financial relationships that could be construed as a potential conflict of interest.
